# Causal effect of serum matrix metalloproteinase levels on venous thromboembolism: a Mendelian randomization study

**DOI:** 10.4178/epih.e2024046

**Published:** 2024-04-07

**Authors:** Deheng Han, Fangcong Yu, Liangrong Zheng

**Affiliations:** Department of Cardiology, The First Affiliated Hospital, Zhejiang University School of Medicine, Hangzhou, China

**Keywords:** Venous thromboembolism, Mendelian randomization analysis, Matrix metalloproteinases, Pulmonary embolism, Venous thrombosis

## Abstract

**OBJECTIVES:**

Serum matrix metalloproteinase (MMP) levels are associated with cardiovascular diseases. However, the causal associations between serum levels of specific MMPs and venous thromboembolism (VTE) remain unclear. The present study sought to explore the causal relationship between serum MMP levels and VTE by using the Mendelian randomization (MR) method.

**METHODS:**

In this study 2-sample MR study, the exposure data on serum MMP levels were derived from genome-wide association studies involving 21,758 individuals from 13 cohorts of European descent. The outcome data on VTE, including deep vein thrombosis and pulmonary embolism, were derived from the FinnGen research project. The primary method used was the inverse-variance weighting method. The MR-Egger intercept test and the Cochran Q test were used to evaluate pleiotropy and heterogeneity.

**RESULTS:**

Using the inverse-variance weighting method, higher serum MMP-12 levels were found to be associated with an increased risk of VTE (odds ratio, 1.04; 95% confidence interval, 1.01 to 1.07; p=0.001). Moreover, there was a weak association between the levels of certain MMPs and VTE. Sensitivity analyses revealed no significant heterogeneity and pleiotropy in our study, and the Steiger directionality test did not reveal a significant reverse causation association.

**CONCLUSIONS:**

There is a causal association between MMP-12 levels and VTE, which may have substantial implications for the diagnostic and therapeutic strategies used for VTE.

## GRAPHICAL ABSTRACT

[Fig f4-epih-46-e2024046]

## Key Message

We found that there is a causal association between matrix metalloproteinase-12 levels and venous thromboembolism. Serum matrix metalloproteinase may have profound implications on the diagnostic andtherapeutic strategies used for venous thromboembolism.

## INTRODUCTION

Venous thromboembolism (VTE) is a multicausal disease that includes pulmonary embolism (PE) and deep vein thrombosis (DVT). It ranks as the third most common cardiovascular disease, affecting nearly 10 million people worldwide annually [1-3]. VTE is associated with both inherited genetic factors and acquired conditions, such as cancer, obesity, surgery, and infection [1,4]. Understanding the pathophysiological mechanisms and risk factors associated with VTE is crucial for its effective prevention, diagnosis, and treatment.

Matrix metalloproteinases (MMPs) belong to the zinc-dependent endopeptidase family and have the ability to degrade virtually all constituents of the extracellular matrix, including elastin, fibronectin, and collagen, thereby promoting tissue repair and regeneration [5,6]. MMPs help maintain the delicate balance between extracellular matrix turnover and homeostasis under physiological conditions. Recent studies have revealed that MMPs can also participate in immune regulation, transcriptional control, and cell signaling [7]. Understanding the intricate biology of MMPs and their regulatory mechanisms has spurred advancements in basic, preclinical, and clinical investigations. Clinical trials have particularly focused on the effectiveness of MMP inhibitors in treating a variety of diseases, including cardiovascular, neurological, oncological, and inflammatory conditions [8-11].

Mendelian randomization (MR) is a statistical method used to evaluate causal relationships by leveraging genetic variants as instrumental variables (IVs) [12]. Using genetic variants as IVs can prevent reverse causation and confounding bias, enabling a more accurate assessment of the causal relationship between exposures and outcomes [13-15].

To our knowledge, relatively few studies have investigated the relationship between MMP levels and VTE. The current study aimed to examine the causal effect of MMP levels on VTE (including PE and DVT). By elucidating the role of MMPs in VTE, we hope to identify novel diagnostic and therapeutic targets that can ultimately improve patient outcomes and reduce the burden of this life-threatening condition.

## MATERIALS AND METHODS

### Study design

**Figure 1.**
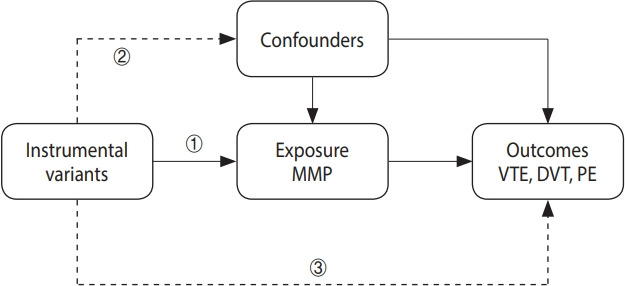
Three core assumptions of the 2-sample Mendelian randomization design in this study. ① The instrumental variable is strongly associated with the serum MMP levels. ② The instrumental variable is independent of confounders. ③ The instrumental variable affects the VTE (including DVT and PE) solely via the serum MMP levels. MMP, matrix metalloproteinase; VTE, venous thromboembolism; DVT, deep vein thrombosis; PE, pulmonary embolism.

This study utilized a 2-sample MR design to explore the causal associations between serum MMP levels and the occurrence of VTE (including PE and DVT). An MR study is based on 3 core assumptions. First, the instrumental variable is strongly associated with the exposure. Second, the instrumental variable is independent of confounders. Third, the instrumental variable affects the outcome solely via the exposure (Figure 1) [13-15].

### Data source

The genetic variants associated with serum MMP levels were identified in a study by Folkersen et al. [16]. This research evaluated 90 candidate biomarkers linked to cardiovascular risk in 21,758 individuals across 1,313 cohorts of European descent. The statistical data are available for download from the SCALLOP CVD-I online resource.

The FinnGen Project is a substantial public-private initiative that aims to collect and analyze genome and health data from 500,000 participants in Finnish biobanks. We extracted summary-level GWAS data for VTE (including DVT and PE) from the FinnGen consortium (Release 9, https://r9.finngen.fi/). This dataset included 19,372 cases and 357,905 controls for VTE (Phenocode: I9_VTE), 9,109 cases and 324,121 controls for DVT (Phenocode: I9_PHLETHROMBDVTLOW), and 9,243 cases and 367,108 controls for PE (Phenocode: I9_PULMEMB). The definitions of VTE, DVT, and PE were based on the International Classification of Diseases, ninth revision.

### Selection of instrumental variables

We selected the single nucleotide polymorphisms (SNPs) to be used as IVs based on the following criteria: First, SNPs were strongly associated with the MMP (p < 5 × 10−8). Second, to prevent weak instrument bias, the F-statistic for each SNP was greater than 10. Third, linkage disequilibrium clumping was used to exclude SNPs in linkage disequilibrium (R^2^ < 0.1 and distance < 10,000 kb) [17,18]. Fourth, the MR pleiotropy residual sum and outlier (MR-PRESSO) test was used to remove potentially pleiotropic SNPs [19]. Fifth, to prevent reverse causality, Steiger filtering was used to identify SNPs indicative of causality in the reverse direction, which were then removed [20,21]. The detailed SNP statistics are presented in the Supplementary Material 1. These include the SNP, sample size, effect allele, other allele, β, standard error, p-value, effect allele frequency (EAF), and the number of SNPs for the exposures and outcomes of all analyses.

### Statistical analysis

The primary method used for this analysis was the random-effect inverse-variance weighting (IVW) method [20]. Complementary methods included the weighted mode, weighted median, MR-Egger, and simple mode methods. The MR-Egger intercept test and the Cochran Q test were utilized to assess horizontal pleiotropy and heterogeneity, respectively [22]. A p-value < 0.05 indicated the presence of horizontal pleiotropy and heterogeneity. We employed the MR-PRESSO method to identify heterogeneous outlier SNPs and to provide a corrected estimate after their removal [23,24].

To prevent reverse causality, Steiger filtering was employed to identify SNPs indicative of causality in the opposite direction. Additionally, the MR-Steiger directionality test was utilized in our analysis [21]. To mitigate the risk of weak instrument bias, the Fstatistic for each SNP was calculated. R^2^ represents the variance explained by each SNP, calculated as R^2^ = 2× (1−EAF)×β^2^ × EAF; F=R^2^/(1−R^2^)× (N−2). Here, β represents the effect size; EAF denotes the EAF; and N is the number of individuals [25,26]. Power calculations were conducted using the online tool mRnd, based on the outcome sample size, proportion of cases, R^2^ sum, and a type I error rate of 0.05 [27,28].

A scatter plot and leave-one-out plot were utilized to visualize the results of our study. Given the multiple analyses conducted, a Bonferroni-corrected p-value of less than 0.0033 (0.05/15) was deemed statistically significant. A p-value between 0.003 and 0.050 was considered suggestive evidence. The analyses were performed using R version 4.2.3 (R Foundation for Statistical Computing, Vienna, Austria) and the TwoSampleMR R package.

### Ethics statement

No patients were directly involved in the overall process of our study. This study was performed based on publicly available data and no separate ethical approval was required.

## RESULTS

### Primary Mendelian randomization analysis

**Figure 2.**
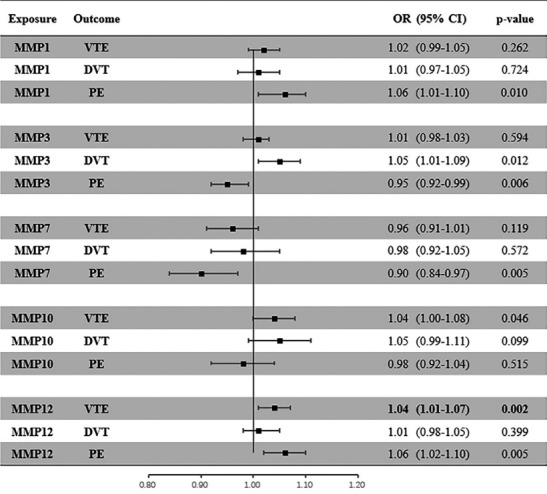
The causal association between MMP and VTE (including DVT and PE) using the inverse-variance weighted Mendelian randomization method. A p-value <0.0033 was considered statistically significant. A p-value ranging from 0.0033 to 0.0500 was regarded as suggestive evidence. OR, odds ratio; CI, confidence interval; MMP, matrix metalloproteinase; VTE, venous thromboembolism; DVT, deep vein thrombosis; PE, pulmonary embolism.

**Figure 3.**
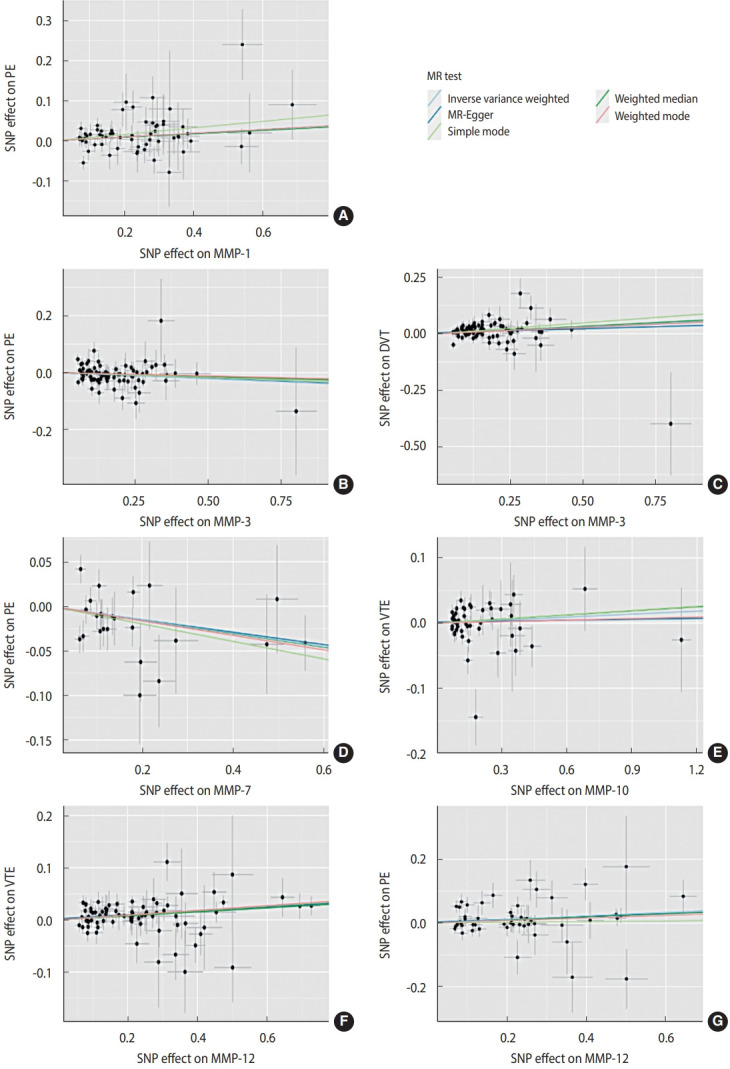
Trends in the causal associations between (A) the MMP-1 level and PE, (B) the MMP-3 level and PE, (C) the MMP-3 level and DVT, (D) the MMP-7 level and PE, (E) the MMP-10 level and VTE, (F) the MMP-12 level and VTE, and (G) the MMP-12 level and PE. MMP, matrix metalloproteinase; VTE, venous thromboembolism; DVT, deep vein thrombosis; PE, pulmonary embolism; MR, Mendelian randomization.

Figure 2 presents the primary results of this MR study using the IVW method. A p-value less than 0.003 was considered statistically significant, while a p-value ranging from 0.003 to 0.050 was regarded as suggestive evidence. Seven trait pairs exhibited statistically significant differences before the Bonferroni correction was applied. Serum MMP-1 levels were associated with an increased risk of PE (odds ratio [OR], 1.06; 95% confidence interval [CI], 1.01 to 1.10; p= 0.010). Serum MMP-3 levels were associated with a higher risk of DVT (OR, 1.05; 95% CI, 1.01 to 1.09; p= 0.012) and a lower risk of PE (OR, 0.95; 95% CI, 0.92 to 0.99; p= 0.006). Serum MMP-7 levels were associated with a lower risk of PE (OR, 0.90; 95% CI, 0.84 to 0.97; p= 0.005). Serum MMP-10 levels were associated with a higher risk of VTE (OR, 1.04; 95% CI, 1.00 to 1.08; p = 0.046). Serum MMP-12 levels were associated with a higher risk of VTE (OR, 1.04; 95% CI, 1.01 to 1.07; p= 0.001) and PE (OR, 1.06; 95% CI, 1.02 to 1.10; p= 0.005). However, after applying the Bonferroni correction, only the association between serum MMP-12 levels and a higher risk of VTE remained significant. The scatter plots of significant MR results before the Bonferroni correction are displayed in Figure 3.

### Sensitivity tests

The weighted mode, MR-Egger, and weighted median methods were used to evaluate the causal association between MMP levels and VTE (including DVT and PE). Although several associations did not show statistical significance, the results were in the same direction as those obtained using the primary IVW method (Supplementary Material 2).

The pleiotropy of the study was evaluated using the MR-Egger regression method, and no significant pleiotropy was detected in our analyses (p for intercept > 0.05). The study’s heterogeneity was assessed with the Cochran Q test, and no significant heterogeneity was observed in our analyses, except for the association between MMP-12 levels and PE (Table 1). Due to the presence of heterogeneity, both the MR-PRESSO outlier test and leave-one-out analysis were conducted to identify and eliminate outlier SNPs (Supplementary Material 3). Additionally, random-effect models were employed in our analysis to reduce the impact of heterogeneity. The MR-Steiger directionality test yielded a result of “true” for all tests, indicating the absence of reverse causal associations.

The statistical power for the main analyses met the 80% threshold, with the exception of the association between MMP-10 and VTE (Supplementary Material 4). This discrepancy may be attributed to the minimal variance in MMP-10 explained by the selected IVs. Therefore, caution is advised when interpreting this particular result. Overall, however, our results are considered relatively reliable.

## DISCUSSION

In our study, we employed the MR method to explore the causal relationship between serum MMP levels and the incidence of VTE, which includes DVT and PE. Following Bonferroni correction, the findings suggested that elevated serum MMP-12 levels are associated with an increased risk of VTE.

To our knowledge, this study is the first to explore the causal relationship between serum MMP levels and VTE. VTE is a disease with multiple causes, including inherited genetic factors and acquired factors. It is also a common complication in patients who are bedridden for extended periods and those who have undergone surgery [1,4]. In clinical practice, D-dimer serves as a crucial laboratory marker for patients at low risk, boasting a sensitivity of up to 95% in diagnosing VTE. However, its specificity is low [29]. Therefore, it is necessary to identify new biomarkers that offer both high sensitivity and specificity.

Some clinical and basic foundational studies have investigated the association between MMPs and thrombi. One study found that the upregulation of MMPs is involved in left atrial appendage thrombus formation in elderly people [30]. Another study demonstrated a disequilibrium of MMPs in the superficial venous wall in patients with superficial venous thrombosis. Therefore, MMPs may be implicated in the pathogenesis of superficial venous thrombosis [31]. Furthermore, in a mouse model of myocardial infarction, increased MMP levels were associated with the formation of intracardiac thrombi [32].

Previous studies also investigated the causal effect of MMP levels on ischemic stroke through MR. The first study examined the causal effects of MMP-1, MMP-8, and MMP-12 levels on ischemic stroke. It found that lower serum levels of MMP-12 were associated with an increased risk of ischemic stroke, lower serum levels of MMP-1 and MMP-12 were linked to an increased risk of large-artery stroke, and higher serum levels of MMP-8 were associated with an increased risk of small vessel stroke [33]. The second Mendelian study focused on the association between MMP-8 levels and ischemic stroke and its subtypes, finding that higher serum levels of MMP-8 were associated with increased risks of small vessel stroke [34]. The third Mendelian study investigated promising therapeutic targets for ischemic stroke identified from plasma and cerebrospinal fluid proteomes. Using different databases, it found that lower serum levels of MMP-12 were associated with an increased risk of ischemic stroke [35]. These 3 MR studies showed relatively consistent results.

However, epidemiological studies investigating the association between MMP-8 levels and the risk of stroke have yielded inconsistent results; several studies have demonstrated a significant correlation [36,37], while others have not [38,39]. There are many reasons for this divergence. First, observational studies suffer from several methodological limitations for causal inference, including inherent biases such as confounding and reverse causation. Second, the databases for stroke in MR studies were mainly from individuals of European descent, whereas the observational cohort was ethnically diverse. Third, the clotting process during serum preparation is known to release MMPs from circulating leukocytes. Therefore, measuring this proteinase from serum also reflects the potential of the neutrophils to degranulate and release it, and this degree may depend on genetic variations [40]. Overall, larger studies are needed to confirm the causal relationships between serum MMP levels and stroke.

Although the precise relationship between MMP-12 levels and VTE is not fully understood, with ongoing research, blood stasis, endothelial damage, and hypercoagulability are recognized as the three primary components of thrombosis [41]. MMP-12 was first identified and characterized in macrophages [42,43]. This macrophage-derived MMP-12 can break down the protein that connects endothelial cells, resulting in cell apoptosis and injury [43]. Additionally, in a model of vascular injury, biomarkers associated with vascular damage were found to be reduced in MMP-12 knockout mice [44].

In addition to its degradative mechanism, MMP-12 promotes inflammatory responses, which are crucial for the development of thrombosis. In mice with MMP-12 overexpression, there is an increase in chemokine secretion and the recruitment of inflammatory cells, such as macrophages, to the sites of inflammation [45]. In MMP-12 knockout mice, the inflammation was comparatively more attenuated than in control mice [46].

Furthermore, MMP-12 has been implicated in the regulation of fibrinolysis, a process in which blood clots are broken down [47]. It inhibits fibrinolysis by degrading plasminogen activators, which are essential for the breakdown of fibrin clots. This inhibition may contribute to the persistence and growth of blood clots in VTE [48,49]. Overall, the relationship between MMP-12 levels and VTE involves vascular injury, modulation of inflammatory responses, and regulation of fibrinolysis. However, further research is needed to fully understand the complex interactions and potential mechanisms involved in this association.

In our analysis, before applying the Bonferroni correction, serum MMP-3 levels were associated with an increased risk of DVT (OR, 1.05; 95% CI, 1.01 to 1.09; p= 0.012) and a decreased risk of PE (OR, 0.95; 95% CI, 0.92 to 0.99; p= 0.006). However, these associations disappeared after the Bonferroni correction was applied. PE comprises a group of clinical syndromes characterized by the obstruction of the pulmonary artery or its branches by various emboli. These emboli can include materials such as thrombus, fat, air, amniotic fluid, bone marrow, metastatic cancer, bacteria, and cardiac organisms. DVT involves the clotting of venous blood within the deep veins of the lower extremities and does not necessarily result in PE. Further research is required to elucidate these discrepancies.

All results presented in this study were based on the IVW method. Various types of sensitivity analysis further confirmed the strength and reliability of our findings. The IVW method is likely to provide the most reliable causal estimates. When exploring the association between MMP-12 levels and VTE, the MR-Egger method (p= 0.087) and the weighted mode method (p= 0.006) appeared to be somewhat inconsistent with the main findings. Although the p-values did not reach statistical significance, the different MR methods (IVW, MR-Egger, and weighted mode) demonstrated directionally consistent results, leading us to consider our results robust.

An advantage of this study is that it employed the MR method to investigate the relationship between MMP levels and VTE, effectively reducing the influence of confounding factors and avoiding erroneous causal associations. The GWAS data for MMPs and VTE were obtained from a large cohort. Furthermore, sensitivity analyses showed no significant horizontal pleiotropy or heterogeneity in our study, and the Steiger directionality test did not indicate any significant reverse causal relationships.

However, our study also had some limitations. First, although the cohort was considerably large, it is important to note that all patients were of European descent. Therefore, the findings may not be applicable to other populations. Second, we investigated only the causal association between five specific MMPs and VTE. The GWAS data for other MMPs were relatively limited, resulting in an extremely small number of SNPs available for analysis. Third, we could not perform a subgroup analysis because the GWAS data consisted only of summary-level statistics. Fourth, the statistical power for the association between MMP-10 and VTE did not reach the 80% threshold, which could be due to the minimal variance in MMP-10 explained by the selected instrumental variables. Therefore, caution is advised when interpreting the results.

In conclusion, this MR study established a causal relationship between MMP-12 levels and VTE, which could significantly impact the diagnostic and therapeutic approaches for VTE. Further research is required to confirm these findings and investigate the underlying mechanisms.

## Figures and Tables

**Figure f4-epih-46-e2024046:**
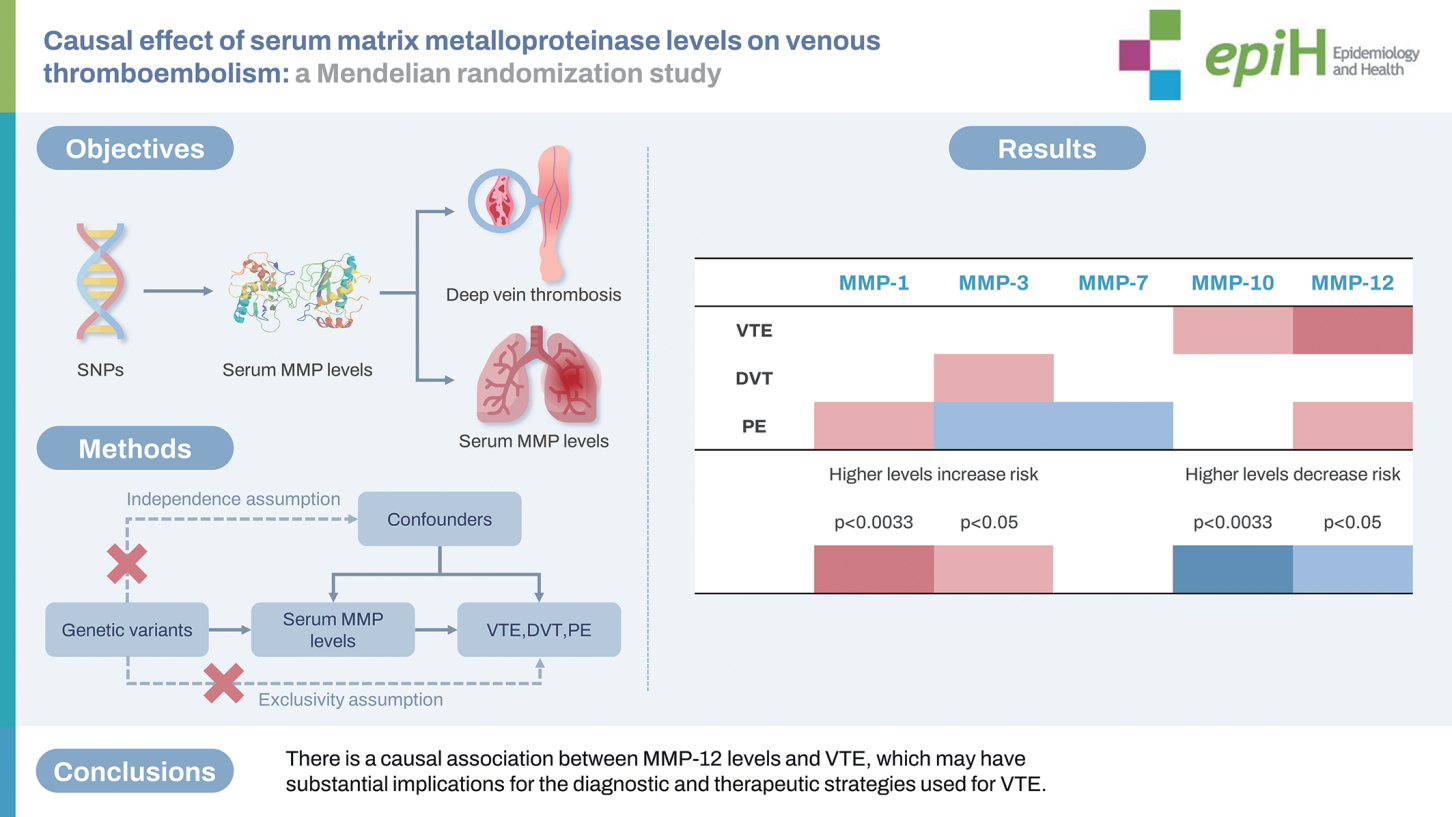


**Table 1.** Heterogeneity and pleiotropy tests of the significance of causal effects of MMPs on VTE

Exposure	Outcome	Pleiotropy			Heterogeneity	
		Intercept	SE	p-value	Q	p-value
MMP1	VTE	-0.005	0.006	0.448	52.521	0.059
MMP1	DVT	0.007	0.009	0.469	54.215	0.053
MMP1	PE	0.004	0.009	0.658	52.579	0.072
MMP3	VTE	0.007	0.005	0.148	61.105	0.208
MMP3	DVT	0.001	0.007	0.841	62.543	0.199
MMP3	PE	-0.002	0.006	0.760	56.594	0.378
MMP7	VTE	-0.001	0.007	0.854	21.751	0.194
MMP7	DVT	0.000	0.009	0.988	11.071	0.853
MMP7	PE	-0.009	0.010	0.367	20.029	0.219
MMP10	VTE	0.003	0.007	0.682	38.413	0.072
MMP10	DVT	0.003	0.009	0.780	36.935	0.120
MMP10	PE	-0.003	0.010	0.741	42.412	0.030
MMP12	VTE	0.000	0.004	0.965	62.097	0.159
MMP12	DVT	-0.001	0.006	0.826	40.464	0.877
MMP12	PE	0.003	0.007	0.722	82.454	0.005

MMP, matrix metalloproteinase; VTE, venous thromboembolism; DVT, deep vein thrombosis; PE, pulmonary embolism; SE, standard error.

## Data Availability

All the datasets used in the present study are publicly available. The data generated or analyzed during this study have been included in this published article.
