# Supplementary material for: Causal effect of serum matrix metalloproteinase levels on venous thromboembolism: a Mendelian randomization study
Source: Epidemiol Health. 2024 Apr 7;46:e2024046. doi: 10.4178/epih.e2024046 (PMC11417446; doi:10.4178/epih.e2024046)
Supplement: Supplementary Material 3. [file epih-46-e2024046-Supplementary-3.docx]

Causal effect of serum matrix metalloproteinase levels on venous thromboembolism: A mendelian randomization Study

**Author information**

Deheng Han

Department of Cardiology, The First Affiliated Hospital, Zhejiang University School of Medicine, Hangzhou, China.

Fangcong Yu

Department of Cardiology, The First Affiliated Hospital, Zhejiang University School of Medicine, Hangzhou, China.

Liangrong Zheng

Department of Cardiology, The First Affiliated Hospital, Zhejiang University School of Medicine, Hangzhou, China.

**Corresponding author**

Liangrong Zheng

Degree: MD. Phd.

Department and affiliation: Department of Cardiology, The First Affiliated Hospital, Zhejiang University School of Medicine, Hangzhou, China.

E-mail address: 1191066@zju.edu.cn

Mailing address: No. 79 Qingchun Road, Hangzhou, Zhejiang Province, China

**
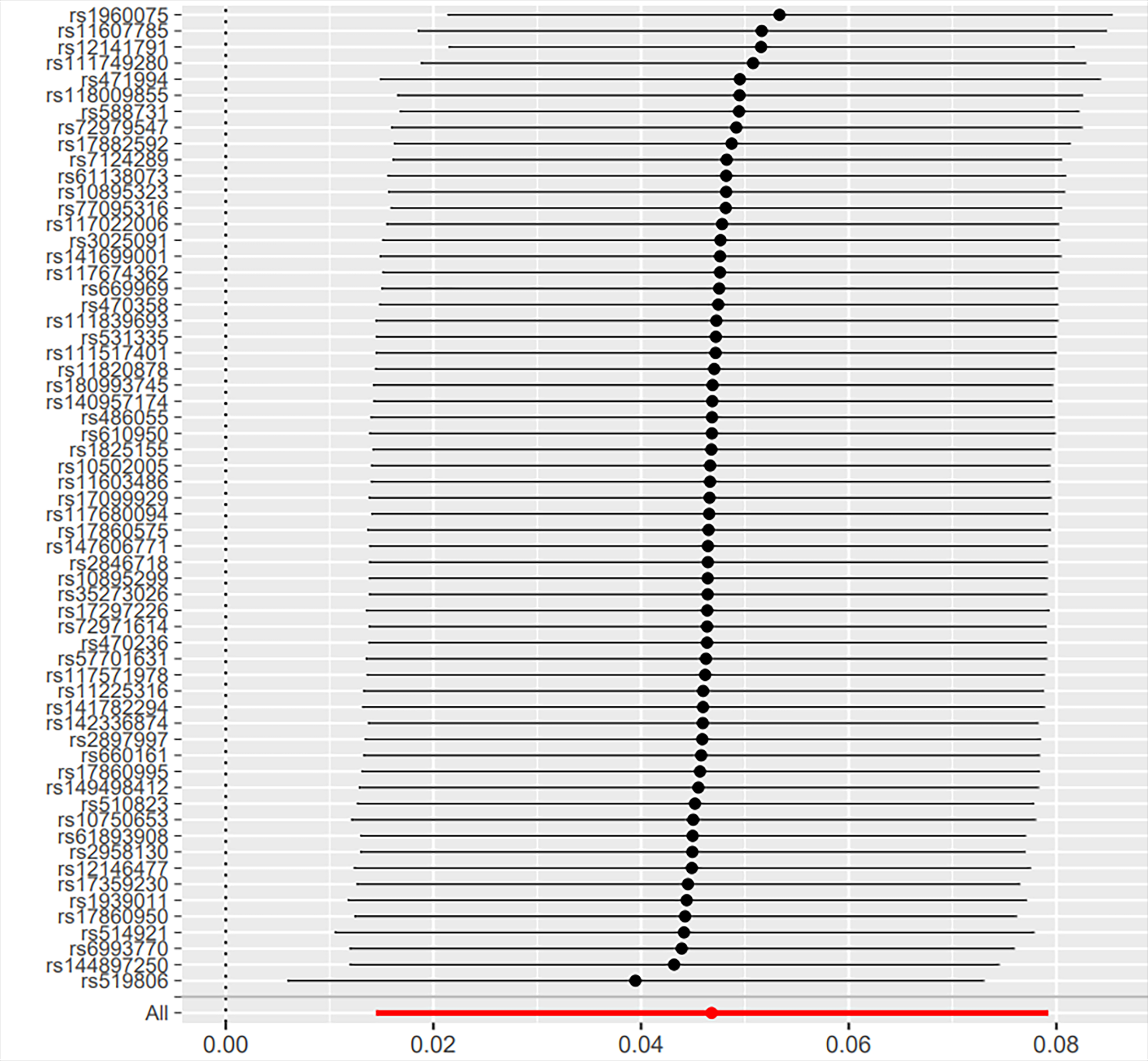
**

**Supplementary Fig. 1** Leave-one-out sensitivity analyses for the associations between MMP-1 and pulmonary embolism

**
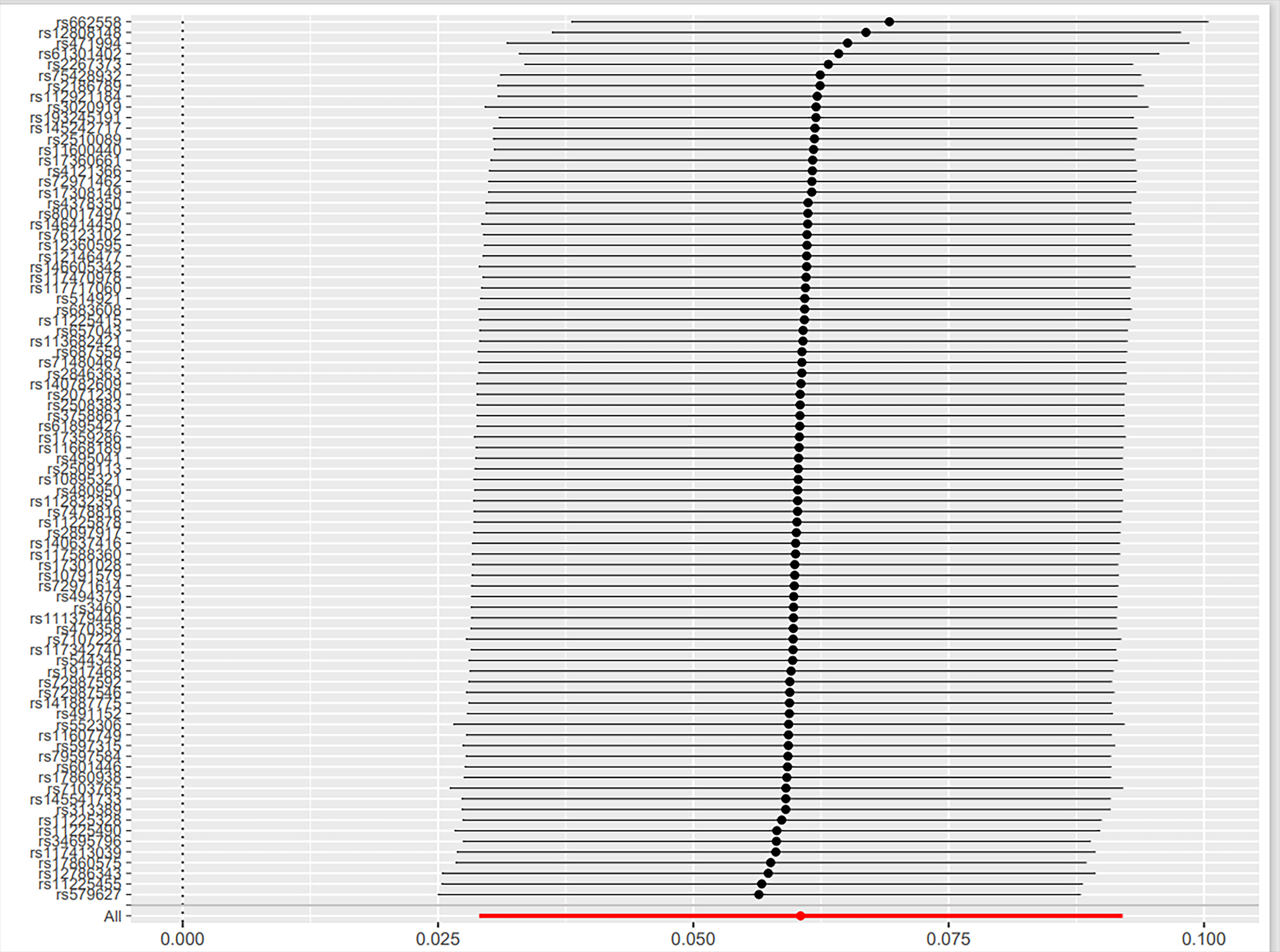
**

**Supplementary Fig. 2** Leave-one-out sensitivity analyses for the associations between MMP-3 and deep vein thrombosis

**
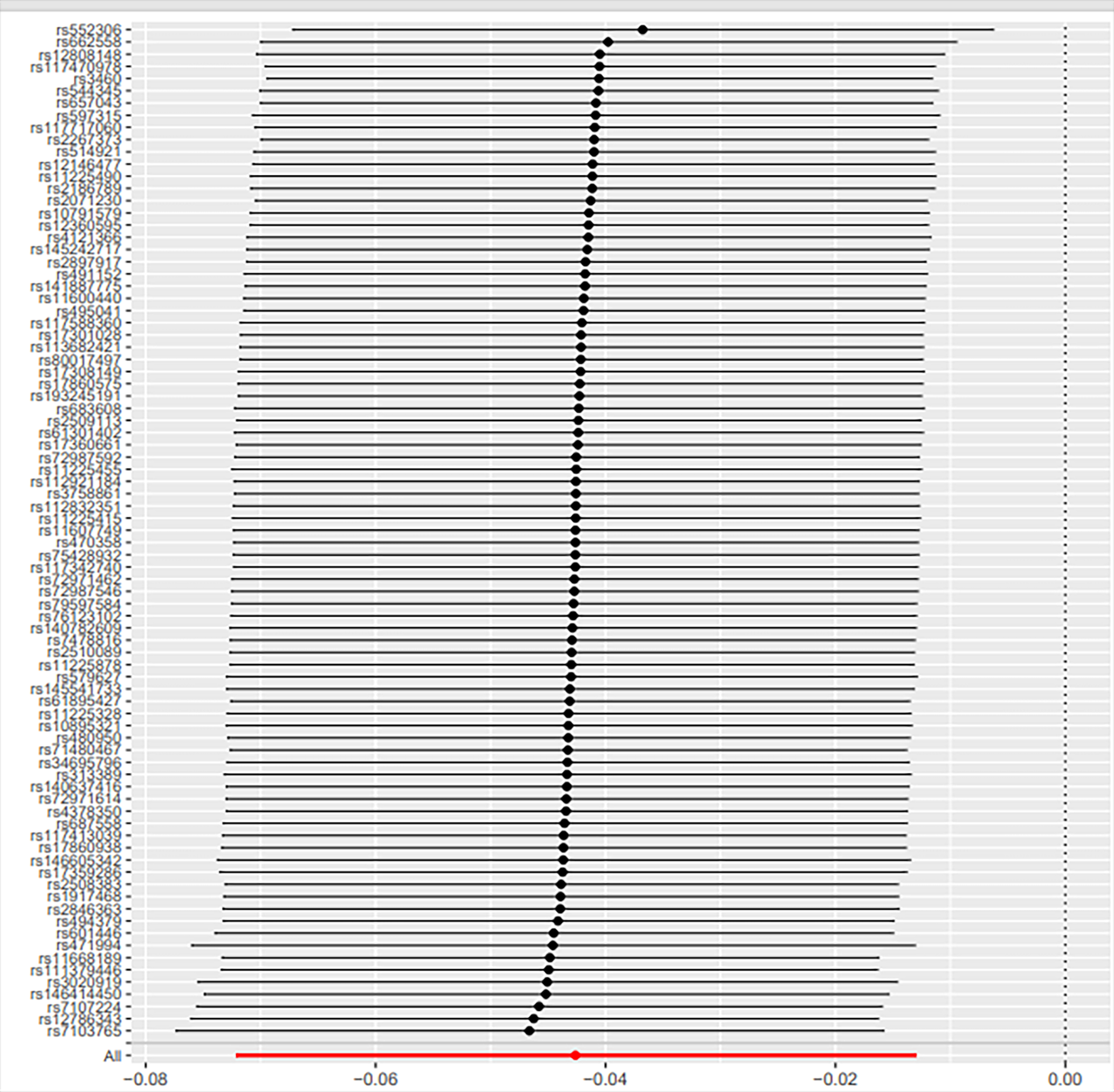
**

**Supplementary Fig. 3** Leave-one-out sensitivity analyses for the associations between MMP-3 and pulmonary embolism

**
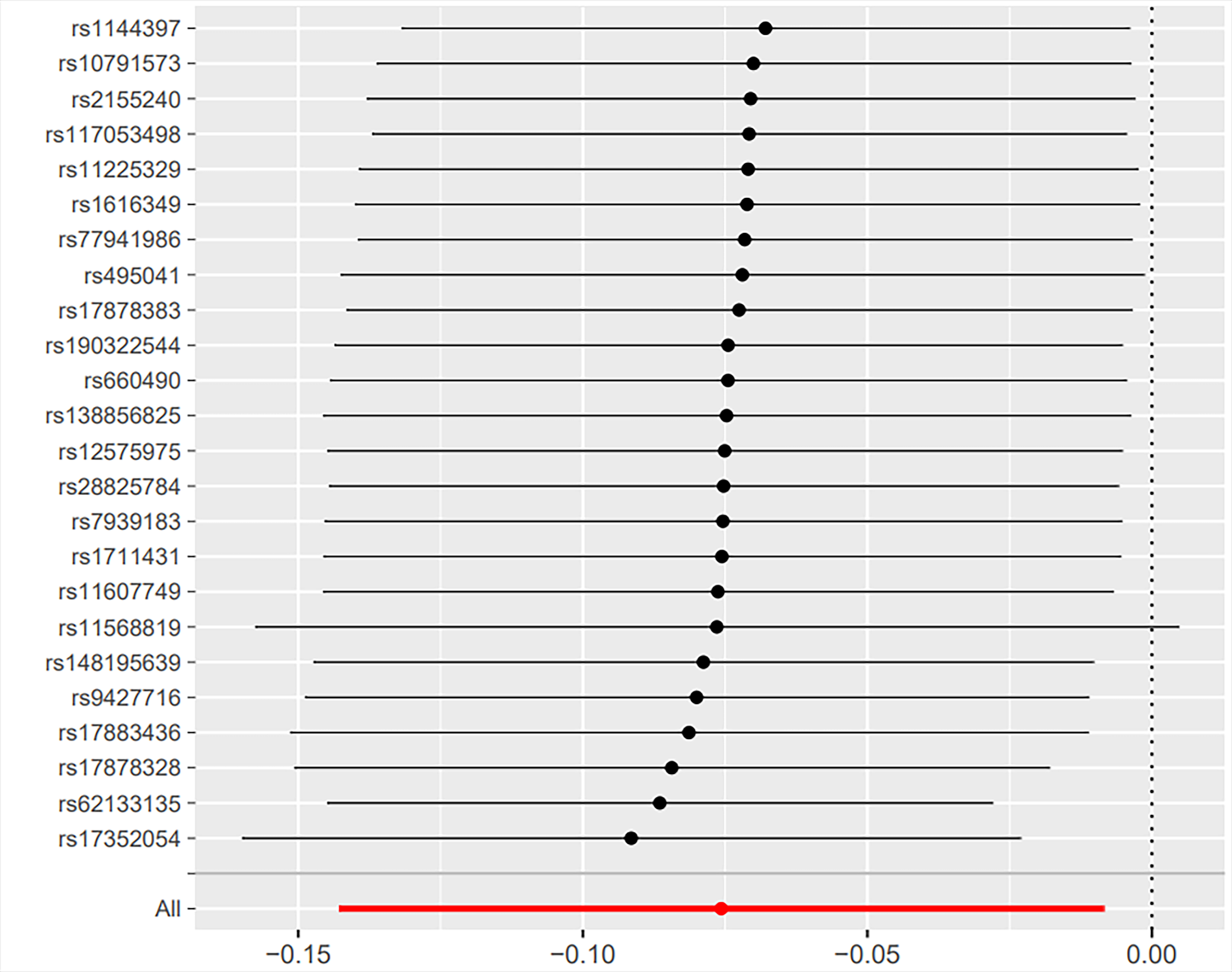
**

**Supplementary Fig. 4** Leave-one-out sensitivity analyses for the associations between MMP-7 and pulmonary embolism

**
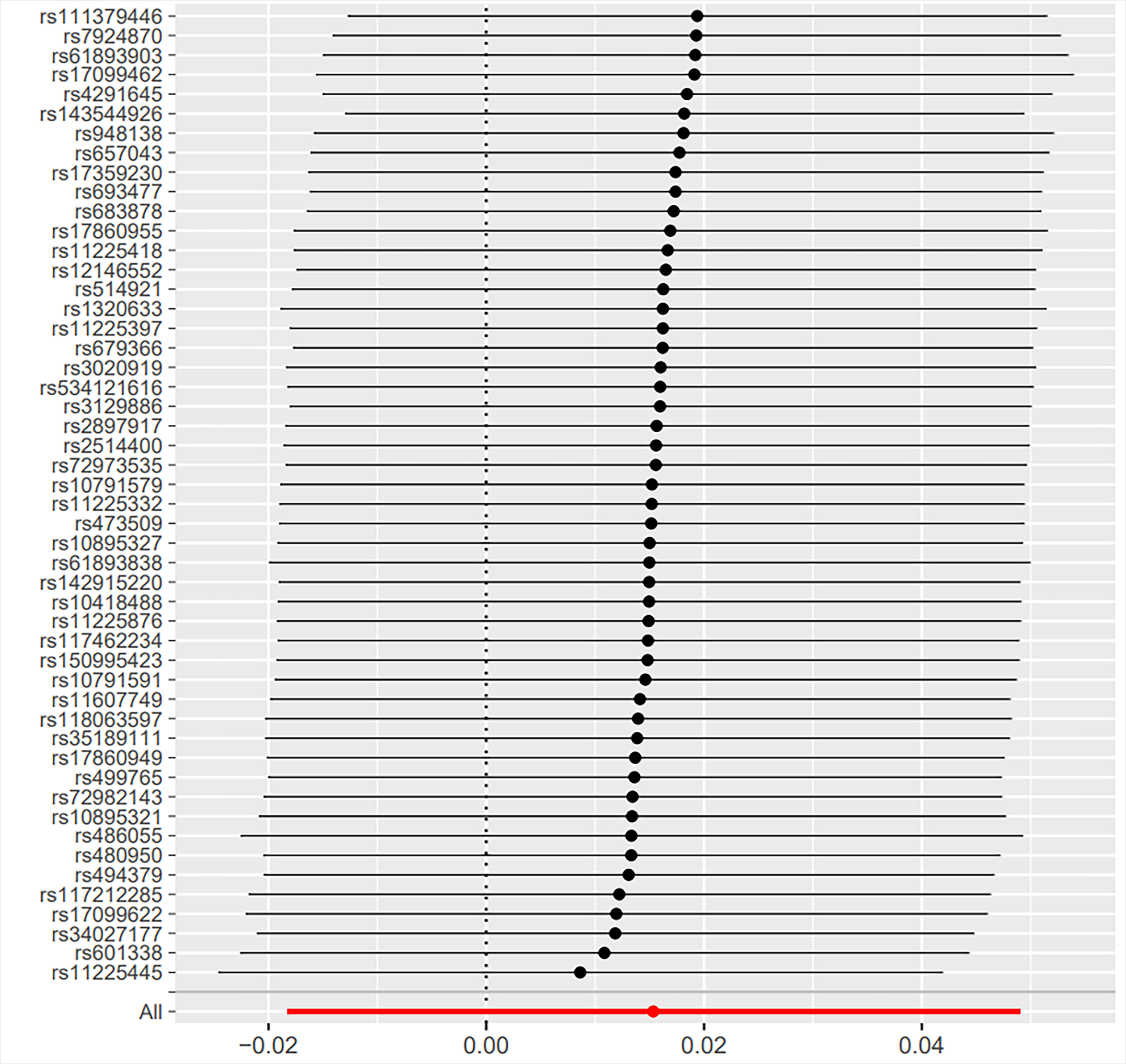
**

**Supplementary Fig. 5** Leave-one-out sensitivity analyses for the associations between MMP-10 and venous thromboembolism

**
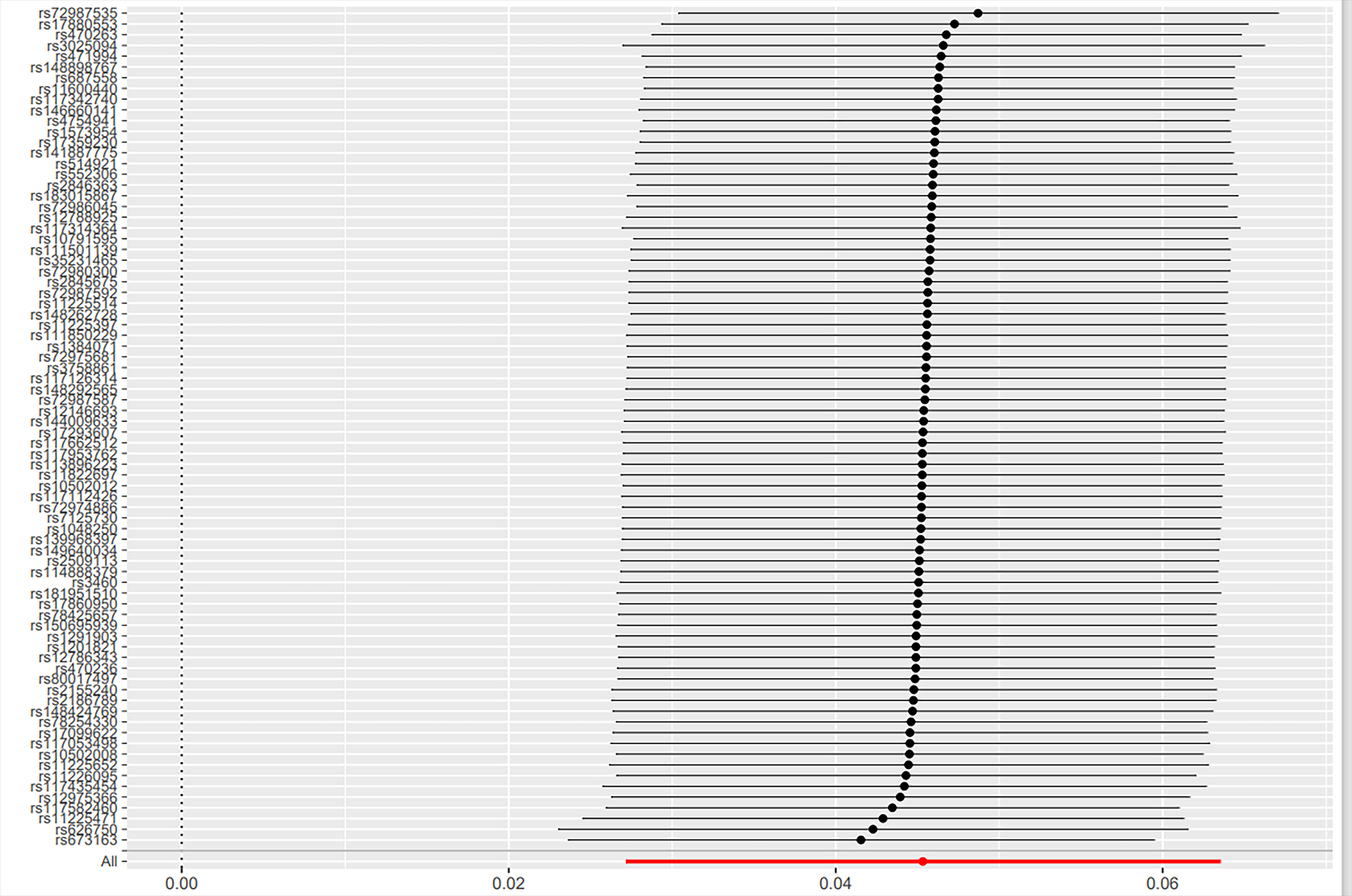
**

**Supplementary Fig. 6** Leave-one-out sensitivity analyses for the associations between MMP-12 and venous thromboembolism

**
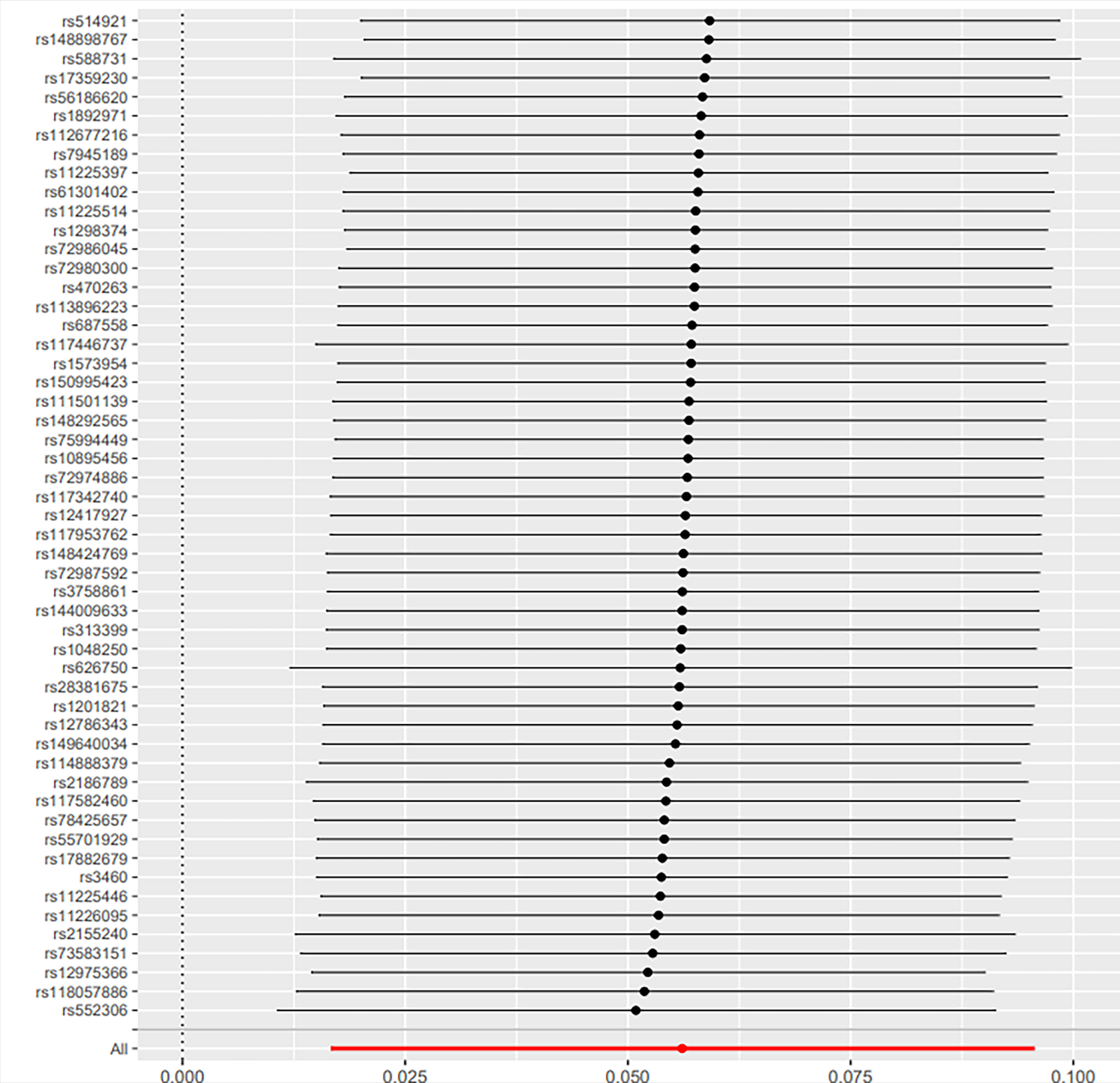
**

**Supplementary Fig. 7** Leave-one-out sensitivity analyses for the associations between MMP-12 and pulmonary embolism
